# Therapeutic potential of hyaluronic acid hydrogel combined with bone marrow stem cells-conditioned medium on arthritic rats’ TMJs

**DOI:** 10.1038/s41598-024-77325-6

**Published:** 2024-11-05

**Authors:** Mai M. Assi, Mohammed E. Grawish, Heba Mahmoud Elsabaa, Mohamad E. Helal, Samah K. Ezzat

**Affiliations:** 1 Department of Oral Biology, Faculty of Dentistry, Mansoura University, Mansoura, 35511 Egypt; 2 Department of Oral Biology, Faculty of Oral and Dental Medicine, Delta University for Science and Technology, Dakahlia, Egypt; 3 Department of Oral Biology and Pathology, Faculty of Dentistry, Badr University in Cairo, Cairo, Egypt

**Keywords:** Hyaluronic acid, Bone marrow stem cells-conditioned medium, Induced arthritis, Temporomandibular joint, Rats, Biological techniques, Stem cells

## Abstract

Conditioned media (CM) is derived from mesenchymal stem cells (MSC) culture and contains biologically active components. CM is easy to handle and reduces inflammation while repairing injured joints. Combination therapy of the CM with cross-linked hyaluronic acid (HA) could ameliorate the beneficial effect of HA in treating degenerative changes of articulating surfaces associated with arthritic rats’ temporomandibular joints (TMJs). This study aimed to evaluate the therapeutic potential of HA hydrogel combined with bone marrow stem cells-conditioned medium (BMSCs-CM) on the articulating surfaces of TMJs associated with complete Freund’s adjuvant (CFA)-induced arthritis. Fifty female Sprague-Dawley rats were divided randomly into five equal groups. Rats of group I served as the negative controls and received intra-articular (IA) injections of 50 µl saline solution, whereas rats of group II were subjected to twice IA injections of 50 µg CFA in 50 µl; on day 1 of the experiment to induce persistent inflammation and on day 14 to induce arthritis. Rats of group III and IV were handled as group II and instead, they received an IA injection of 50 µl HA hydrogel and 50 µl of BMSCs-CM, respectively. Rats of group V were given combined IA injections of 50 µl HA hydrogel and BMSCs-CM. All rats were euthanized after the 4th week of inducing arthritis. The joints were processed for sectioning and histological staining using hematoxylin and eosin, Masson’s trichrome and toluidine blue special staining, and immunohistochemical staining for nuclear factor-kappa B (NF-κB). SPSS software was used to analyze the data and one-way analysis of variance followed by post-hoc Tukey statistical tests were used to test the statistical significance at 0.05 for alpha and 0.2 for beta. In the pooled BMSC-CM, 197.14 pg/ml of platelet-derived growth factor and 112.22 pg/ml of interleukin-10 were detected. Compared to TMJs of groups III and IV, TMJs of group V showed significant improvements (*P* = 0.001) in all parameters tested as the disc thickness was decreased (331.79 ± 0.73), the fibrocartilaginous layer was broadened (0.96 ± 0.04), and the amount of the trabecular bone was distinctive (19.35 ± 1.07). The mean values for the collagen amount were increased (12.29 ± 1.38) whereas the mean values for the NF-κB expression were decreased (0.62 ± 0.15). Combination therapy of HA hydrogel and BMSCs-CM is better than using HA hydrogel or BMSCs-CM, separately to repair degenerative changes in rats’ TMJs associated with CFA-induced arthritis.

## Introduction

Arthritis affects millions of people worldwide, with an increasing prevalence due to aging^1^. The prevalence of rheumatoid arthritis (RA) is 0.5-1% in the general population, and it is more frequent in women^2^. The frequency of clinical temporomandibular joints (TMJs) involvement in patients with RA ranges from 5 to 86%, with bilateral involvement reported as the most frequent^3^. Izawa et al.^4^ reported the prevalence of TMJ osteoarthritis (OA) by magnetic resonance imaging and clinical examinations, estimating 70% in the 73–75 years age group and 25% prevalence in the 20–49 years age group. Arthritis is a chronic or acute joint inflammation that often coexists with structural damage and pain. Arthritis of more than 100 different types has been described, the most common ones are RA and OA^5^. They are both inflammatory joint diseases that involve synovial and joint destruction, immune cell infiltration accompanied by joint swelling, pain, and limited movement. These devastating effects reduce the quality of life, increase dependency on certain medications, and decline the physical function which makes even the simplest tasks seem challenging^6^.

The primary goal in the treatment of arthritis is to find therapies that allow patients to move their joints without pain. Treatments of arthritis could include physical or occupational therapy, hot or cold compresses, joint immobilization, massage and exercise, transcutaneous electrical nerve stimulation, acupuncture, and drugs. Nonsteroidal anti-inflammatory and corticosteroid medications only control symptoms and delay injury but cannot reverse the progression of arthritis. Thus, end-stage degenerative joint diseases may require arthroplasty and osteotomy, but also these procedures can result in serious complications such as prosthetic infection, thromboembolism, pain, and postoperative stiffness^7^. Viscosupplementation with intra-articular (IA) hyaluronic acid (HA) hydrogel injection is a well-established treatment option to contrast joint damage, improve joint function, and reduce pain^8^.

Hyaluronic acid (known as sodium hyaluronate or hyaluronan) is a linear non-sulfated polysaccharide consisting of alternately repeating D-glucuronic and D-N-acetylglucosamine units. HA exists naturally in all vertebrates of humans and animals beings, and almost originated in all body tissues and fluids, such as eye vitreous humor, synovial fluid, and hyaline cartilage^9^. There are several varieties of HA with different molecular weights; low (500–730 kDa), intermediate (800–2000 kDa), and high (2000–6000 kDa), including cross-linked formulations of HA^10^. HA is involved in many key processes including pathobiology, matrix organization, morphogenesis, tissue regeneration, wound reparation, and cell signaling, in addition, it has unique physico-chemical properties, such as viscoelasticity, hygroscopicity, mucoadhesivity, biodegradability, and biocompatibility^11^. The FDA premarket approval database revealed no post-marketing reports concerning unexpected adverse events of HA^12^.

Using mesenchymal stem cells-conditioned medium (MSCs-CM) in regenerative medicine is still in its early stages. MSCs-CM reduces disease severity and immune responses in inflammatory arthritis^13^, has a beneficial effect in enhancing processes associated with chondrocyte OA pathomechanism^14^ and represents a new regenerative treatment in several musculoskeletal pathologies^15^. Emerging evidence suggests that MSCs exert effects by generating a wide range of bioactive factors. The factors referred to as CM consists of exosomes, ectosomes, lipid mediators, cell adhesion molecules, chemokines, cytokines, hormones, and growth factors^16^. CM may provide significant benefits over cells in handling, preservation, manufacture, potential as a ready-to-use biologic product, and longevity of the product^17^. As there is a lack of evidence about the beneficial impact of MSC-CM on both immunological and clinical outcomes, and before clinical trials, animal models should be used in pre-clinical research with prolonged observation periods^18^.

Clinically, IA injection of HA for joint arthritis has been widely studied. Concentrated growth factor combined with sodium hyaluronate was effective in TMJ OA treatment^19^. IA injection of platelet rich plasma with HA following arthrocentesis had a synergistic effect in reducing pain and improving function in TMJ OA^20^. CM from stem cells of human exfoliated deciduous teeth alleviates mouse OA by inducing secreted frizzled-related protein 1-Expressing anti-inflammatory M2 macrophages in the synovium^21^. Combined therapy of HA and BMSCs-CM has not been investigated clinically or experimentally on animal models as a treatment option for OA. Only one study evaluated and compared the anti‑inflammatory effect of non‑animal stabilized HA and MSCs-CM in an explant‑based coculture model of OA. The finding indicated that treatments with this combination therapy could be a therapeutic option that may help counteract the catabolism produced by the inflammatory state in knee OA^22^.

There is no treatment that cures arthritis and therefore development of therapeutic alternatives that can prevent the destruction of the joint structures or stimulate its adequate repair is required. Since the combination therapy of HA hydrogel with bone marrow stem cells BMSCs-CM could produce a more effective treatment for arthritis, this study aimed at evaluating the rational use of HA hydrogel combined with BMSCs-CM on the articulating surfaces associated with complete Freund’s adjuvant (CFA)-induced arthritis in rats’ TMJs through using IA injections. The null hypothesis was that the combination therapy of HA hydrogel and BMSCs-CM would have the same effect as HA hydrogel or BMSCs-CM when used separately.

## Materials and methods

### Animals

Fifty, male healthy Sprague-Dawley rats, eight weeks old, weighing 150–200 g were purchased from the Medical Research Centre at Faculty of Medicine, Mansoura University. In a light-controlled room with a 12:12 h light-dark cycle and a temperature of 22 °C, rats were kept in separate cages. The relative humidity was maintained between 65 and 70%. The rats were free to roam and were given water and commercial soft diet. Regarding use and care of animals and for this experiment, the Institutional Animal Care and Use Committee of the Faculty of Dentistry, Mansoura University, Egypt (A14060421), approved this study. The guidelines of the Animal Research: Reporting In Vivo Experiments and the ARRIVE Checklist (https://www.nc3rs.org.uk/arrive-guidelines) were followed in performing this study and all methods were performed in accordance with the American Veterinary Medical Association guidelines.

### Chemicals

1. Complete Freund’s adjuvant (Sigma Aldrich, Saint Louis, Missouri, USA). Each 1 mL consists of 1 mg of heat-killed and dried *mycobacterium tuberculosis*, 0.85 mL paraffin oil, and 0.15 mL mannide monooleate^23^.
2. Hyaluronic acid hydrogel (Crespine^®^ Gel, GmbH & Co. KG, Dümmer, Germany). The gel is a cross-linked hyaluronic acid, sterile, viscoelastic, biocompatible, resorbable IA implant of high purity. It contains 1.0 mg hyaluronic acid, 14.0 mg hyaluronic acid cross-linked, 6.9 mg sodium chloride and 1.0 ml water for injection^24^.
3. Enzyme-linked immunosorbent assay (ELISA) Kits (CAT# CSB-E04595r, Cusabio Biotech Co, Wuhan, China) to measure the amount of platelet-derived growth factor (PDGF) and interleukin-10 (IL10) cytokines in the samples of the BMSC-CM.

### Study design

The sample size was calculated using G* Power 3.1.9.2 software. The statistical test was ANOVA (Fixed effects, special, main effects, and interactions) and the test family was F test. The type of power analysis was to estimate the required sample size given the following; power, affect size, and α. The input parameters were effect size, 0.50; power (1-β error probability), 0.80; and α error probability, 0.15. Notably, 5% is commonly used by researchers as a cutoff p-value to determine the significance level. However, the 15% for α is acceptable depending on cost/benefit ratio of the research^25,26^ The number of groups was 5 and the numerator degree of freedom was 10. The total sample size was 50. The rats were divided randomly into five equal groups using a random-numbers table, 10 rats each. The left TMJ of each rat was used only in this experiment to avoid hindering the chewing ability and feeding. Rats of group I served as the negative controls, and received IA injections of 50 µl saline, whereas rats of group II were subjected to twice IA injections of 50 µg CFA in 50 µl; on day 1 of the experiment to induce persistent inflammation and at day 14 to induce arthritis^27,28^ Rats of groups III and IV were handled as group II and instead, they received an IA injection of 50 µl HA hydrogel^29^. and 50 µl of the BMSCs-CM, respectively^30^. Rats of group V were given a combined IA injection of 50 µl HA hydrogel and BMSCs-CM^31^. All rats were euthanized after the 4th week of inducing arthritis.

### Experimental induction of arthritis

All rats were anesthetized by an intraperitoneal infusion of 25 mg/kg xylazine and 75 mg/kg ketamine. The skin surrounding the TMJ was shaved and was cleaned carefully with 70% ethyl alcohol, and then the postero-inferior border of the zygomatic arch was palpated before IA injections. A 26-gauge needle was inserted underneath this location and was advanced anteriorly and medially contacting with the condyle. The needle’s penetration into the joint space was verified by the loss of resistance after the mandible was moved to confirm this contact. Aspiration was cautiously ruled out before the administration of CFA to avoid intravascular implantation. The suspension was frequently disseminated throughout the articular area by repeatedly extending and flexing the joints^32^. The animals were monitored daily for the onset of arthritis for 22–28 days^28^. In accordance to the Osteoarthritis Cartilage Histopathology Assessment System proposed by Pritzker et al.,^33^ [OA Research Society International (OARSI)], the OA scoring was estimated according to two parameters; the grade and stage assessing cartilage pathogenesis semi-quantitatively by measuring both the vertical grade (0–6 points) and horizontal stage (0–4 points) progression of OA cartilage manifestations. The overall score is defined as the combined assessment of OA severity and extent 0–24.

### Preparation and collection of BMSCs-CM

A cryopreserved rat cell line with cell density 1 × 10^6^ from the Nile Centre for Experimental Research, Mansoura, Egypt, was used to produce BMSCs-CM. At passage 3 and after BMSCs reached 70–80% confluence, the Gibco Dulbecco’s Modified Eagle Medium/Nutrient Mixture F-12 (Thermo Fisher Scientific, USA) supplemented with 10% fetal bovine serum was removed. The cells were washed thrice with phosphate buffer saline (PBS), and the culture medium was replaced with serum-free minimal essential medium (Thermo Fisher Scientific, USA). Then, the culture flasks were incubated for 72 h before collecting and centrifuging the medium at 1500 rpm for 5 min at 4 ^o^C and the supernatants were collected and centrifuged again at 3000 rpm for 3 min at 4 ^o^C. The resultant supernatants were filtered through a 0.22- µm filter unit and kept in tiny tubes at -80 ^o^C until they were needed in the study^34,35^ These procedures were completed aseptically using class II A2, UNIL@B biological safety cabinet.

### ELISA assay of BMSCs-CM

According to the manufacturer’s instructions, ELISA was performed using the pooled BMSC-CM to determine the concentrations of PDGF and IL10. The quantitative enzyme immunoassay technique was used with specific antibodies for the target PDGF or IL10 proteins. The antibodies had been pre-coated onto a microplate (100 µL). Standards and samples were pipetted into the wells and the immobilized antibody bound any target protein present. After removing any unbound substances, a biotin-conjugated antibody specific for PDGF or IL10 was added to the wells. After washing, avidin-conjugated horseradish peroxidase was added to the wells. Following a wash to remove any unbound avidin-enzyme reagent, a substrate solution was added to the wells, and color was developed in proportion to the amount of protein bound in the initial step. The color development was stopped by adding a stop solution and the intensity of the color was measured using a microplate reader capable of measuring absorbance at 450 nm with the correction wavelength set at 540–570 nm. The quantitative concentration results of PDGF and IL10 were plotted and were compared to a standard curve. The signal was proportional to the concentration of the target and detected after the addition of a substrate solution. The detection range for PDGF was 7.8–500 pg/ml whereas for IL10 was 3.12–200 pg/ml. The minimum detectable dose of rat PDGF-AB was typically less than 1.95 pg/ml whereas for IL10 was typically less than 0.78 pg/ml. The sensitivity of this assay or the lower detection was defined as the lowest protein concentration that could be differentiated from zero.

### BMSCs-CM and HA hydrogel injection

HA hydrogel and BMSCs-CM were mixed with a ratio of 1:1 and were shaken by vortex until homogenization to form BMSCs-CM/ HA hydrogel^35^. After inducing arthritis, rats of groups III, IV, and V received respectively a single dose of 50 µl HA hydrogel, 50 µl BMSCs-CM, and 50 µl of HA hydrogel combined with BMSCs-CM via IA injection into the arthritic TMJs using a needle after anesthesia^28^. The IA administration of various therapeutics was applied in a similar way to CFA injection and all rats were euthanized after 4 weeks from inducing arthritis.

### Histological examination

At the end of the experiment, each rat was euthanized by intraperitoneal injection of 200 mg/kg sodium pentobarbital euthanasia solution. Each rat was decapitated and its TMJs were removed, and processed for paraffin blocks. The specimens were put in 10% formaldehyde for 24 h. Following fixation, the specimens were rinsed under running water to get rid of any residual fixative. Then, they were decalcified in 10% ethylene diamine tetra acetic acid disodium salt solution of pH 7, for 3–4 weeks. The completeness of decalcification endpoint was determined through mechanical testing by needling in a remote part away from TMJs. An automatic tissue processor was used to prepare the tissue sections as the specimens were dried using a series of progressive alcohol baths before clearing them with xylene. The specimens were infiltrated and were embedded with melted paraffin. Using a microtome, cutting was performed at 4–6 μm thickness. Serial sagittal sections of the TMJ were performed with every fifth section stained with hematoxylin and eosin (H&E) as a routine one, a sixth section for Masson’s trichrome as special staining, and a seventh section for immunohistochemical (IHC) staining for nuclear factor-kappa B (NF-κB) and eighth section for toluidine blue staining. Two histopathologists blindly examined each section.

### Digital morphometric analyses

The histomorphometry was performed for the stained sections, and 10 images for each group were assessed. 40 X objective, 1/2 X picture adaptor, and Olympus^®^ digital camera mounted on an Olympus^®^ microscope (CX23, Wuhan Aliroad Medical Equipment Co., Ltd, Hubei, China) were used for capturing the images. For Masson’s trichrome and NF-κB, the resulting photos were assessed using Video Test Morphology^®^ software (Russia), which has a particular built-in routine for the area, % area measuring, item counting, and contact angle. A u-tech^®^ frame grabber was used to acquire images from the camera. Depending on the hue of target areas, the color tones of images were enhanced. The images were thresholded at the level of the desired hue range for creating a binary mask representing target areas. The region of interest (ROI) was established for the binary mask. To calculate the percentage area of ROI in relation to the overall field area, a calculating technique known as percentage area (%) calculating routine was used. While for articular disc thickness (µm), fibrocartilaginous layer thickness (µm) and amount of trabecular bone (mm^2^), Image J 1.37v (National Institutes of Health, Bethesda, MD; http://rsb.info.nih.gov/ij/) was used. Two blinded examiners separately measured thickness of the articular disc for anterior band, intermediate zone, and posterior band^27^, and thickness of the fibrocartilaginous layer was measured as the surface of the condylar cartilage was divided equally into three parts. The thickness of the fibrocartilage was measured at the quartering points of the posterior third of the condylar cartilage^36^ and for the trabecular bone, the scale was set as 300 pixels equal to 1 mm^2^, and a separate surface area was bordered using the polygon selection tool for measurement^28^.

### Statistical analyses

Data were tabulated, coded, and analyzed using the computer program SPSS (statistical package for social research) version 17.0. Descriptive statistics of mean and standard deviation (SD) were created from the data. A one-way analysis of variance (ANOVA) was performed to compare data for more than two sets of numerical parametric data, and the post-hoc Tukey test was employed for pairwise comparison. Statistical significance was defined at 0.05 for alpha and 0.2 for beta. To determine the effect size and clinical relevancy between the different groups, Cohen’s d statistical test was used by calculating the mean difference between each two groups, and then dividing the result by the pooled SD.

## Results

### ELISA results

The PDGF and IL10 were detected in the pooled MSC-CM. High quantities of PDGF and IL10 were detected, 197.14 pg/ml and 112.22 pg/ml, respectively.

### H&E histological results

**Fig. 1:**
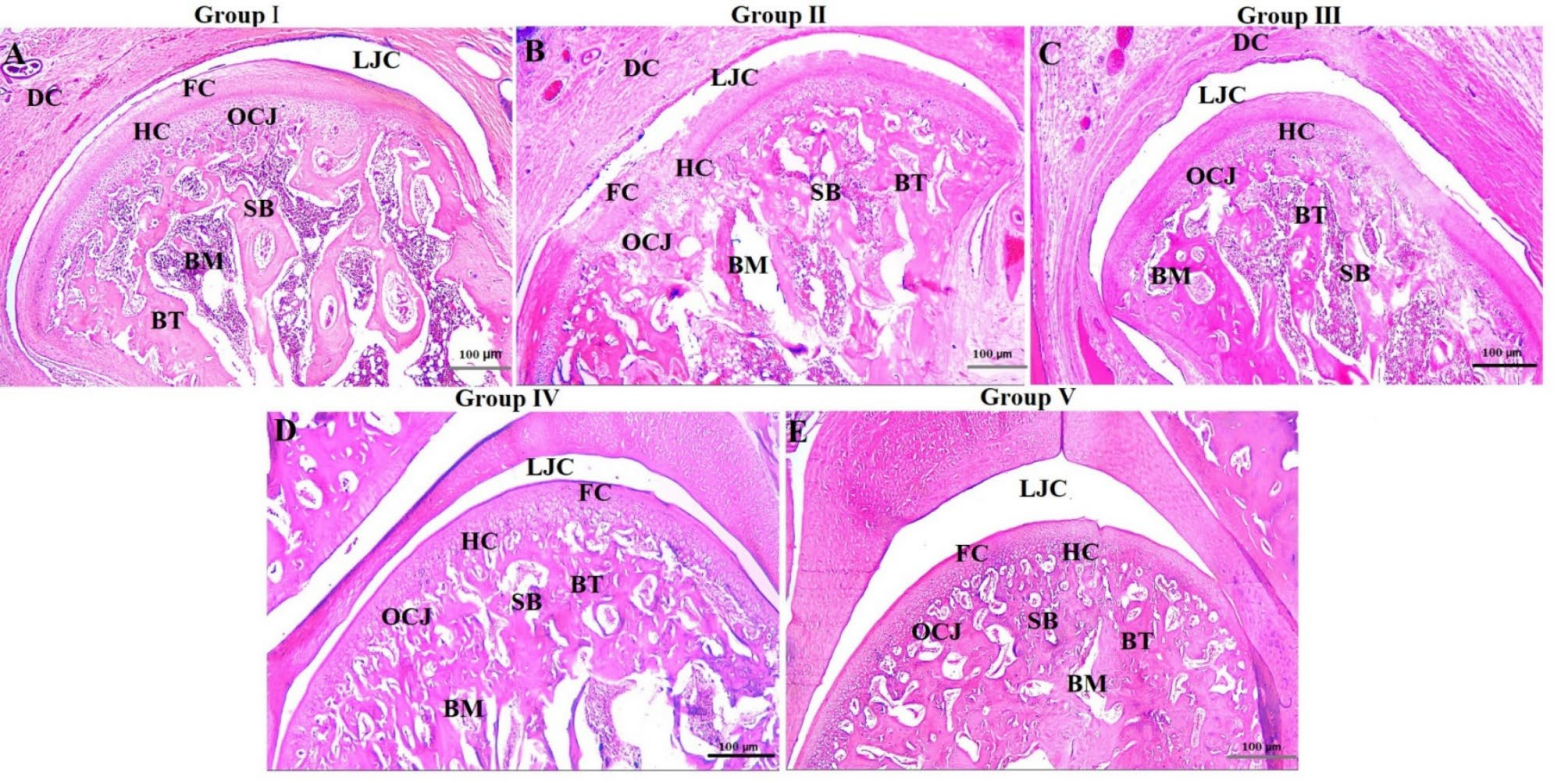
H&E stained representative decalcified TMJ sagittal sections showing (**A**) control group (I), (**B**) arthritic group (II), (**C**) group treated with HA hydrogel (III), (**D**) group treated with BMSCs-CM (IV) and (**E**) group treated with the combination therapy (V). DC; articular disc, LJC; lower joint cavity, FC; fibrocartilaginous layer, HC; hyaline cartilage, OCJ; osteochondral junction, SB; subchondral bone, BT; bone trabeculae, BM; bone marrow, scale bar = 100 μm.

The TMJ’s articular surfaces of group I (negative control) showed normal architectures as they had rounded intact and smooth contours without any soft tissue damages or clefts. The fibrocartilaginous disc was of normal thickness as it had thick posterior and anterior bands separated by a thinner intermediate zone. The condyle consisted of a hyaline cartilaginous plate covered by a superficial layer of dense fibrous connective tissue that includes fibrocartilaginous areas. The resting zone of hyaline cartilage showed a cellular region of small-flattened chondroprogenitor cells, the proliferative one had chondrocytes arranged in columns that were axially orientated to the surface, the hypertrophic zone contained mature and enlarged chondrocytes of spherical shape and, the zone of calcified cartilage passed into the subchondral cancellous bone. The trabeculae of the cancellous bone were of normal thickness, radiating from the center of the condyle reaching the surface at right angles, and they were surrounded by red bone marrow spaces. The interface between the subchondral bone and the cartilaginous plate was assumed by a continuous and regular osteochondral junction (Fig. 1A).

By contrast, group II sections displayed diffuse narrowing of the upper temporodiscal and lower condylodiscal cavities, flattening and roughening of the articulating surfaces, erosions in the condylar fibrocartilaginous layer with the exposure of subchondral bone in certain areas and, a significant articular disc thickening with fibrosis. The articular cartilaginous plate was severely destructed and the clear boundaries between cartilage layers were lost. The cartilaginous layer showed cellular disarrangement with chondrocyte damage and loss. The subchondral bone was severely disrupted, and it had widened marrow spaces accompanied by the resorption of trabecular bone. No clear boundaries were observed between the cartilaginous plate and subchondral bone. There was a flattening of the articular eminence, hyperplastic synovial tissue, and inflammatory mononuclear cell infiltration. (Fig. 1B).

Regarding TMJs of group III, the histological sections revealed a considerable improvement in the structural components of the joint. There was a little decrease in articular disc thickness with a slight increase in the thickness of the condylar fibrocartilaginous layer. The subchondral bone showed more or less normal arrangement and thickness of bone trabeculae **(**Fig. 1C). However, the histological sections of group IV showed marked improvements in the structural components of the joint. The articulating surfaces of both the mandibular condyle and glenoid fossa were regular and smooth. The articular disc was decreased in thickness and became less fibrous, whereas the fibrocartilaginous layer of the condylar head was increased in thickness. The cartilaginous plate showed less damage as the erosion and degeneration were markedly reduced and there was a rise in condylar cartilage thickness with the arrangement of the chondrocytes in a more regular form. By reducing bone resorption, the articular eminence and subchondral bone displayed enhancements in the trabecular connectivity and bone quality. The synovial membrane exhibited a more or less normal histological manner with reduced inflammatory cells (Fig. 1D).

Moreover, TMJs of group V showed significant improvements in TMJ structural components when compared to arthritic, HA hydrogel-treated, and BMSCs-CM-treated groups. TMJs revealed a regain and clearly defined structural elements of the articular disc, condyle, articular eminence, and synovial membrane. Histologically, the subchondral bone and cartilage layer did not show any signs of degenerative alterations. The typical radiating trabeculae that give the subchondral bone its fan-like appearance were present. (Fig. 1E).

### Histomorphometric results

**Fig. 2:**
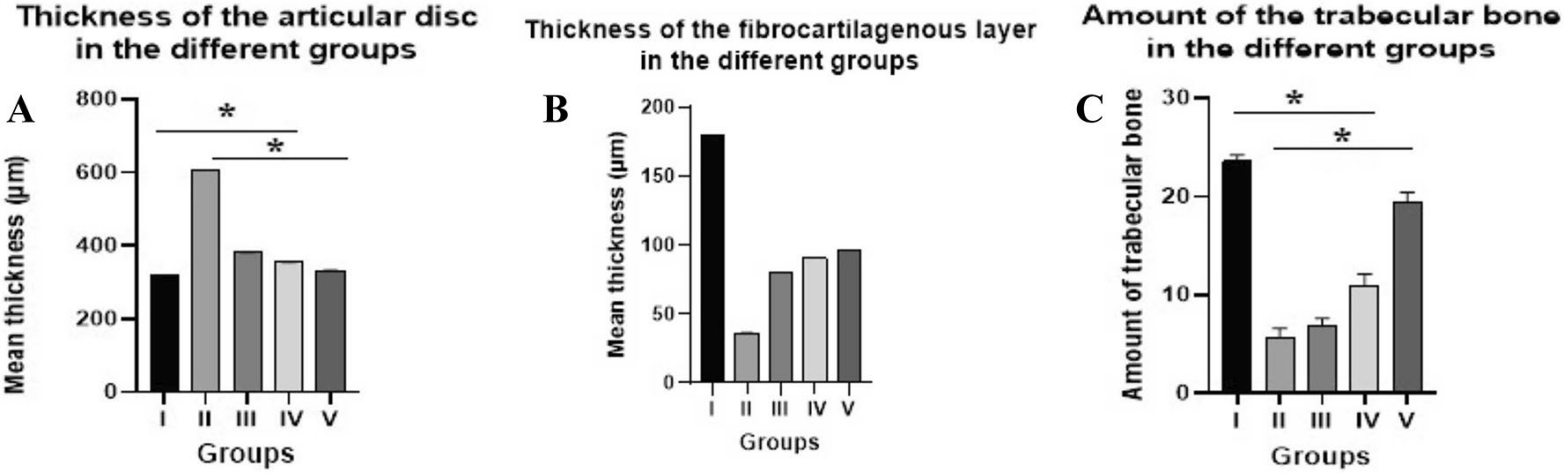
Bar charts showing (**A**) the articular disc thickness, (**B**) fibrocartilaginous layer thickness and (**C**) amount of trabecular bone in the different groups. Data are presented as mean ± SD (one-way analysis of variance with Tukey’s post hoc test). The symbol (*) represents significant difference between different groups.

Regarding disc thickness, the mean value was the highest in group II (606.35 ± 0.67) whereas the lowest was for group I (318.85 ± 0.78). The mean values for the disc thickness for groups III, IV, and V were 381.57 ± 0.76, 354.57 ± 0.53, and 331.79 ± 0.73, respectively. ANOVA test revealed a total significant difference between all groups (*P* = 0.001). Tukey’s post-hoc test revealed significant differences between group I and groups II, III and IV, between group II and groups III, IV and V, between group III and groups IV and V, and between group IV and group V (*P* = 0.001) and non-significant difference between group I and group V (*P* = 0.091). Among the treated groups, the effect size between groups V and I was very small (d = 17.12) and non-significant (*P* = 0.091), very high between groups V and II (d = 391.86) with a significance (*P* = 0.001), moderate between groups V and III (d = 66.80) with a significance and small between groups V and IV (d = 35.71) with a significance (*P* = 0.001) **(**Table 1; Fig. 2A**)**.

**Table 1:** Results of the mean values ± SD for the articular disc thickness (µm) their statistical significance.

Groups	Articular disc thickness				
	Mean ± SD	Cohen’s d, Tukey’s post-hoc test			
		Cohen’s d, P1	Cohen’s d, P2	Cohen’s d, P3	Cohen’s d, P4
Group I	318.85 ± 0.78				
Group II	606.35 ± 0.67	395.41, 0.001			
Group III	381.57 ± 0.76	81.44, 0.001	313.75, 0.001		
Group IV	354.57 ± 0.53	53.56, 0.001	416.80, 0.001	41.21, 0.001	
Group V	331.79 ± 0.73	17.12, 0.091	391.86, 0.001	66.80, 0.001	35.71, 0.001
ANOVA test (F ratio, P value)	(282.2, 0.001)				

*P*1: compared to group I, *P*2: compared to group II, *P*3: compared to group III, *P*4: compared to group IV.

Meanwhile, the mean value of the fibrocartilaginous layer was the highest in group I (179.66 ± 0.15) whereas the lowest was for group II (36.32 ± 0.04). The mean values for the fibrocartilaginous layer thickness for groups III, IV, and V were 80.45 ± 0.04, 90.71 ± 0. 01, and 96.38 ± 0.04, respectively. ANOVA test revealed a total significant difference between all groups (*P* = 0.001). Tukey’s post-hoc test revealed significant differences between group I and groups II, III and IV, between group II and groups III, IV and V, between group III and groups IV and V, and between group IV and group V (*P* = 0.001) and significant difference between group I and group V (*P* = 0.008). Among the treated groups, the effect size between groups V and I was very small (d = 75.66) and significant (*P* = 0.008), very high between groups V and II (d = 1501.50) with a significance (*P* = 0.001), moderate between groups V and III (d = 398.25) with a significance and small between groups V and IV (d = 194.47) with a significance (*P* = 0.001) (Table 2; Fig. 2B).

**Table 2:** Results of the mean values ± SD for the fibrocartilaginous layer thickness (µm) with their statistical significance.

Groups	Fibrocartilaginous layer thickness					
	Mean ± SD	Cohen’s d, Tukey’s post-hoc test				
		Cohen’s d, P1	Cohen’s d, P2	Cohen’s d, P3	Cohen’s d, P4	
Group I	179.66 ± 0.15					
Group II	36.32 ± 0.04	1305.79, 0.001				
Group III	80.45 ± 0.04	903.77, 0.001	1103.25, 0.001			
Group IV	90.71 ± 0.01	836.77, 0.001	1865.56, 0.001	351.91, 0.006		
Group V	96.38 ± 0.04	75.66, 0.008	1501.50, 0.001	398.25, 0.001	194.47, 0.001	
ANOVA test (F ratio, P value)	(440.7, 0.001)					

*P*1: compared to group I, *P*2: compared to group II, *P*3: compared to group III, *P*4: compared to group IV.

Moreover, the mean value of the trabecular bone was the highest in group I (23.56 ± 0.66) whereas the lowest one was for group II (5.68 ± 0.92). The mean values for groups III, IV, and V were 6.79 ± 0.80, 10.89 ± 1.24, and 19.35 ± 1.07, respectively. ANOVA test revealed a total significant difference between all groups (*P* = 0.001). Tukey’s post-hoc test revealed significant differences between group I and groups II, III, and IV, between group II and groups III, IV and V, between group III and groups IV and V, and between group IV and group V (*P* = 0.001) and non-significant difference between group I and group V (*P* = 0.066). Among the treated groups, the effect size between groups V and I was very small (d = 4.73) and non-significant (*P* = 0.066), very high between groups V and II (d = 13.69) with a significance (*P* = 0.001), moderate between groups V and III (d = 10.29) with a significance and small between groups V and IV (d = 7.30) with a significance (*P* = 0.001) (Table 3; Fig. 2C).

**Table 3:** Results of the mean values ± SD for the amount of trabecular bone (mm2) with their statistical significance.

Groups	Amount of trabecular bone				
	Mean ± SD	Cohen’s d, Tukey’s post-hoc test			
		Cohen’s d, P1	Cohen’s d, P2	Cohen’s d, P3	Cohen’s d, P4
Group I	23.56 ± 0.66				
Group II	5.68 ± 0.92	22.33, 0.001			
Group III	6.79 ± 0.80	22.86, 0.001	1.28, 0.001		
Group IV	10.89 ± 1.24	12.75, 0.001	4.77, 0.001	3.92, 0.001	
Group V	19.35 ± 1.07	4.73, 0.066	13.69, 0.001	10.29, 0.001	7.30, 0.001
ANOVA test (F ratio, P value)	(668.5, 0.001)				

*P*1: compared to group I, *P*2: compared to group II, *P*3: compared to group III, *P*4: compared to group IV.

### Trichrome histochemical results

**Fig. 3:**
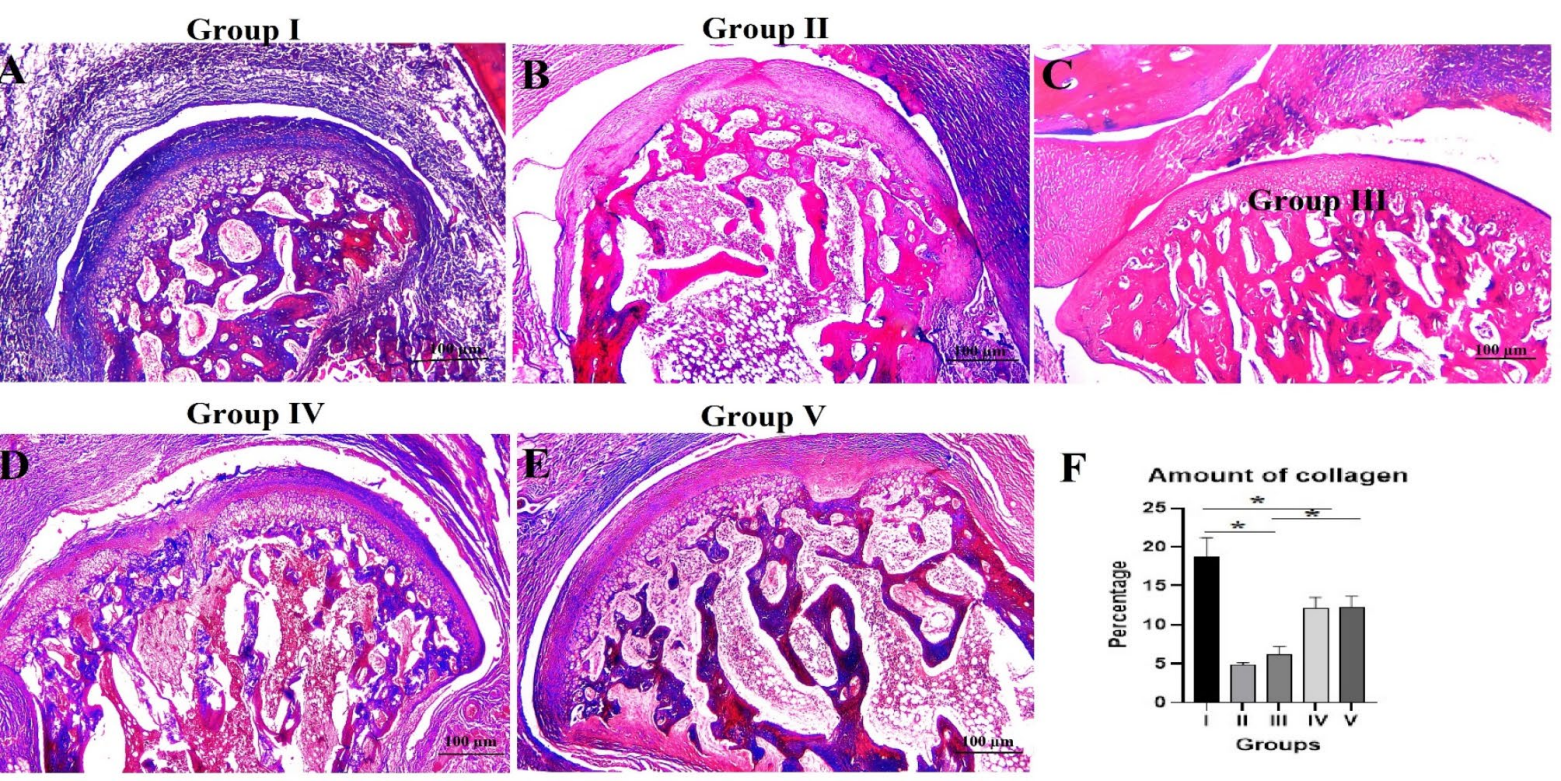
Masson’s trichrome stained representative decalcified TMJ sagittal sections showing (**A**) control group (I), (**B**) arthritic group (II), (**C**) group treated with HA hydrogel (III), (**D**) group treated with BMSCs-CM (IV), (**E**) group treated with the combination therapy (V), scale bar = 100 μm. (**F**) bar chart histogram for the amount of collagen in the different groups, data are presented as mean ± SD (one-way analysis of variance with Tukey’s post hoc test). The symbols (*) represent significant difference between different groups.

The collagen fibers in the fibrocartilage stained blue and the matrix in the hyaline cartilage appeared pink or light purple. Unmineralized new bone and mineralized old trabecular bone were stained blue and red, respectively. Group I sections showed that the collagen fibers were homogeneously distributed within the articular disc. The collagen fibers and chondrocytes of the condylar cartilage were regularly arranged with an ordered trabecular bone structure (Fig. 3A). Meanwhile, in group II sections, the amount of collagen fibers was decreased and their distribution was organized in haphazardly. The diminishing and abnormalities in the trabecular bone structures were accompanied by the small and erratic amount of collagen fibers, indicating remodeling. (Fig. 3B). Group III sections exhibited a relative increase for fibers accompanied with a moderately smooth trabecular bone morphology and a decline in bone marrow cavities (Fig. 3C). Relative to group III, group IV sections showed more improvement in the quantity and arrangement of collagen fibers with a smooth surface of trabecular bone and more shrinkage of bone marrow cavities (Fig. 3D). Whereas group V sections showed a normal pattern of collagen fibers and trabecular bone (Fig. 3E).

The mean value of collagen amount (%) was the highest (18.83 ± 2.30) in group I whereas the lowest one was for group II (4.87 ± 0.25). The mean values for the collagen amount for groups III, IV, and V were 6.14 ± 1.05, 12.21 ± 1.32, and 12.29 ± 1.38, respectively. ANOVA test revealed a total significant difference between all groups (*P* = 0.001). Tukey’s post-hoc test revealed significant differences between group I and groups II, III and IV, between group II and groups IV and V, between group III and groups IV and V (*P* = 0.001), and non-significant difference between group I and group V (*P* = 0.091), between group II and group III (*P* = 0.053) and between group IV and V (*P* = 0.09) (Table 4; Fig. 3F).

**Table 4:** Results of the mean values ± SD for the amount of collagen (%) and expression level of NF-B (%) with their statistical significance.

Groups	Amount of collagen					Expression of NF-kB				
	Mean ± SD	Tukey’s post-hoc test				Mean ± SD	Tukey’s post-hoc test			
		P1	P2	P3	P4		P1	P2	P3	P4
Group I	18.83 ± 2.30					0.36 ± 0.18				
Group II	4.87 ± 0.25	0.001				5.29 ± 0.35	0.001			
Group III	6.14 ± 1.05	0.001	0.053			3.68 ± 0.24	0.001	0.007		
Group IV	12.21 ± 1.32	0.001	0.001	0.001		2.11 ± 0.19	0.004	0.001	0.008	
Group V	12.29 ± 1.38	0.091	0.001	0.001	0.09	0.62 ± 0.15	0.648	0.001	0.001	0.012
ANOVA test (F ratio, P value)	(155.33, 0.001)					(26.79, 0.001)				

*P*1: compared to group I, *P*2: compared to group II, *P*3: compared to group III, *P*4: compared to group IV.

### Toluidine blue histochemical results

**Fig. 4:**
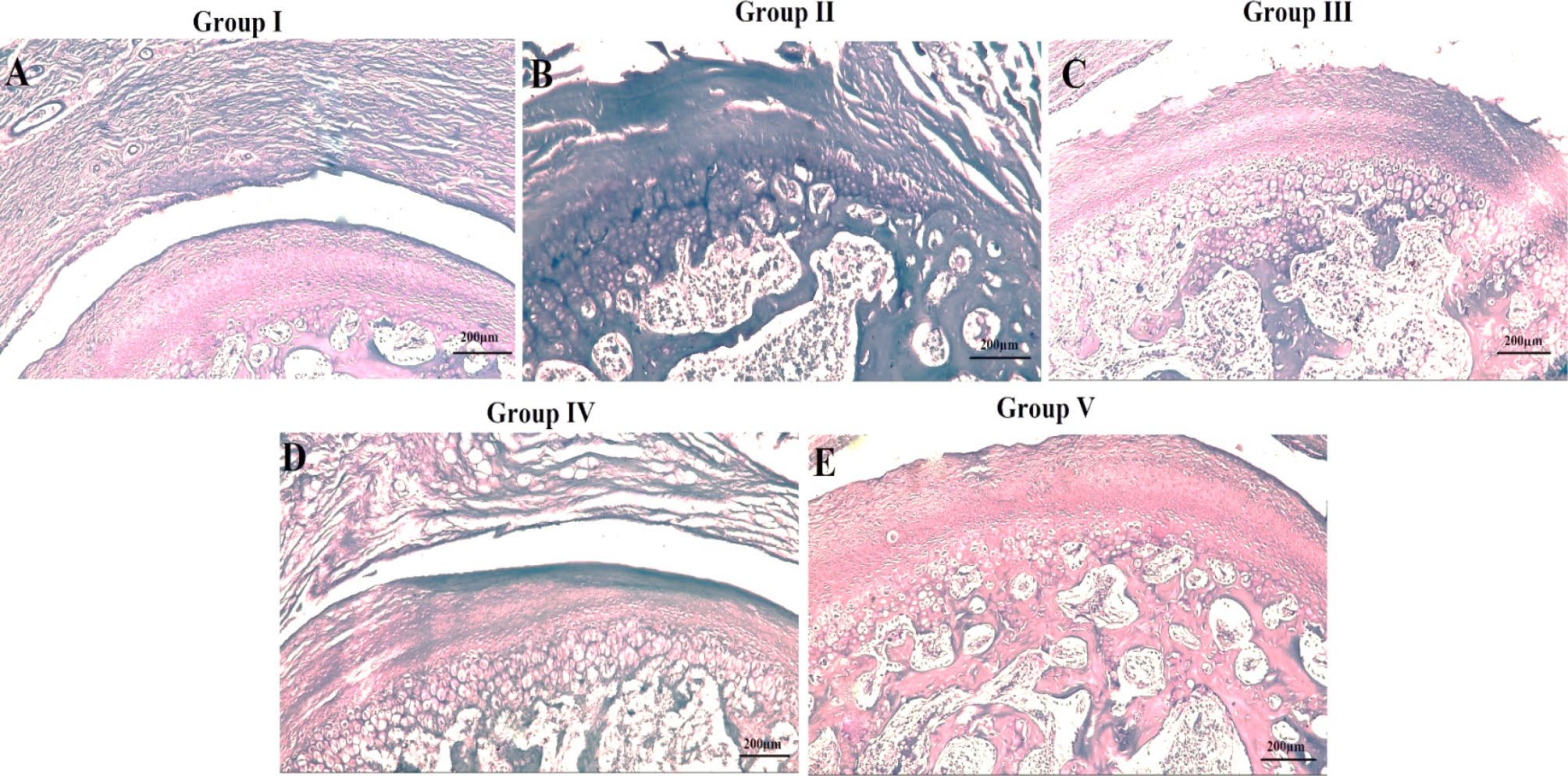
Toluidine blue stained representative decalcified TMJ sagittal sections showing (**A**) control group (I), (**B**) arthritic group (II), (C) group treated with HA hydrogel (III), (**D**) group treated with BMSCs-CM (IV), (**E**) group treated with the combination therapy (V), scale bar = 200 μm.

Toluidine blue (TBO) assessed metachromatic staining of cartilage matrix for increased content of both proteoglycan and glycosaminoglycan production. The polysaccharides of the cartilage stained purple and nuclei were stained blue. Proteoglycan and glycosaminoglycan as part of the extracellular cartilage matrix were evenly distributed in group I (Fig. 4A). Arthritic TMJs of group II showed a marked decrease in proteoglycan and glycosaminoglycan deposition with a pronounced decrease of TBO staining in the cartilaginous areas (Fig. 4B). Meanwhile, arthritic TMJs of group III showed a moderate increase in the amount of proteoglycan and glycosaminoglycan in the disc and fibrous layer covering the articulating surfaces (Fig. 4C) whereas those of group IV revealed a mild increase in the amount of glycosaminoglycan (Fig. 4D). Group V showed an intense TBO staining in the cartilaginous matrix, indicating abundant proteoglycan and glycosaminoglycan deposition similar to group I (Fig. 4E). Assessing 10 images for each group, the OARSI score was 100% grade 0, stage 0 for group I indicating the absence of any arthritic features. For group II, the arthritic score was 90% grade 6, stage 4 and 10% grade 5, stage 4, that is the highest among the studied groups. Meanwhile group III showed arthritic score of 80% grade 2, stage 4 and 20% grade 2, stage 3. Group IV showed arthritic score of 70% grade 3, stage 4 and 30% grade 2, stage 4. Group V recorded a lower level of arthritic score than that recorded in groups III and IV as 90% was grade 1, stage 2 and 10% grade 1, stage 1.

### NF-κB immunohistochemical results

**Fig. 5:**
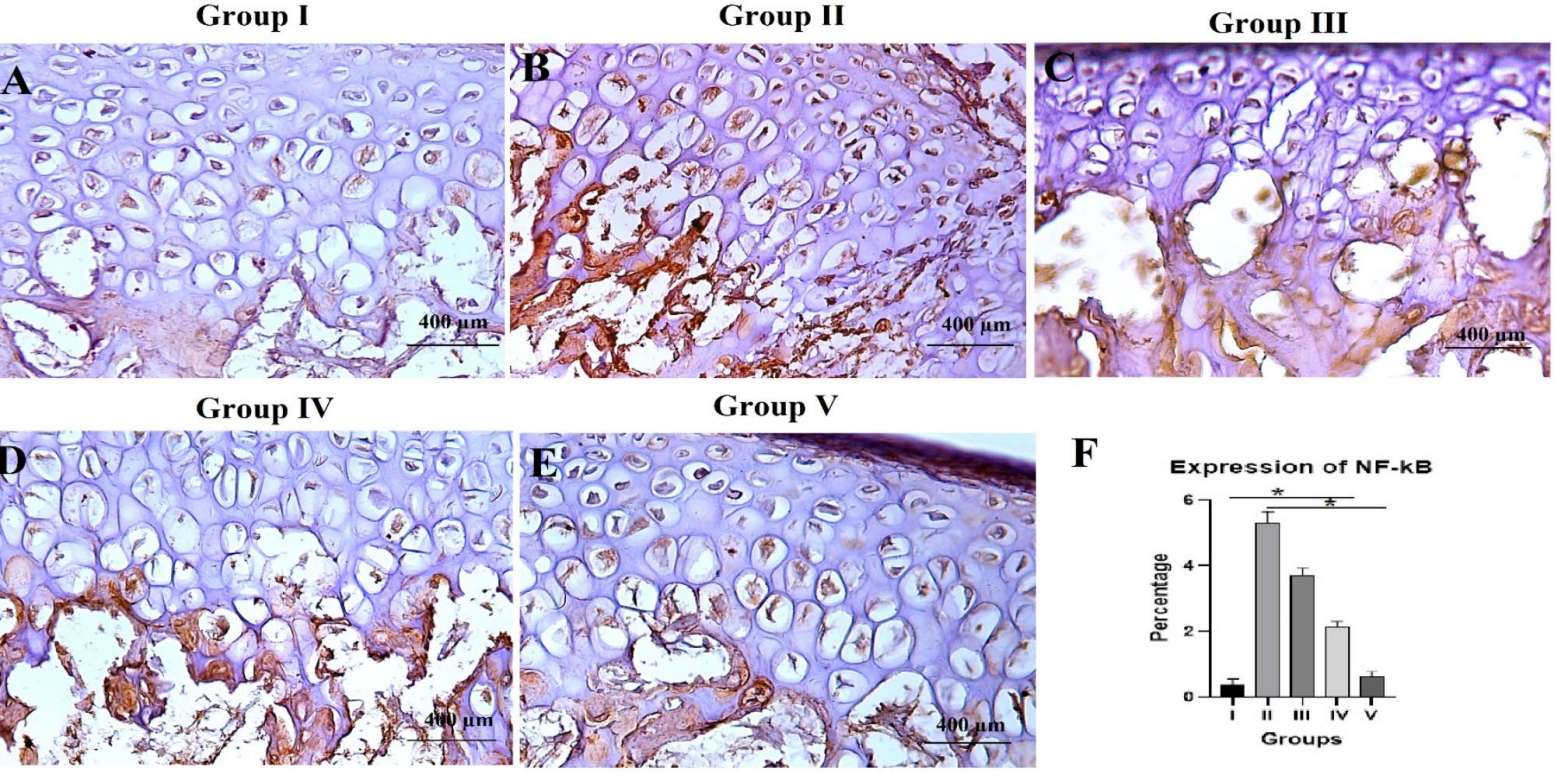
Representative decalcified TMJ sagittal sections showing (**A**) control group (I), (**B**) arthritic group (II), (**C**) group treated with HA hydrogel (III), (**D**) group treated with BMSCs-CM (IV), (**E**) group treated with the combination therapy (V), DAB, scale bar = 400 μm. (**F**) bar chart histogram for the amount of NF-κB expression in the different groups, data are presented as mean ± SD (one-way analysis of variance with Tukey’s post hoc test). The symbol (*) represents significant difference between different groups.

Brown deposits in the cytoplasm were an indication of an immunohistochemical positive reaction. An immune stain was applied to better examine the inflammatory impact of NF-κB. The immune reaction was very mild in group I (Fig. 5A) and severe in group II (Fig. 5B) and it was decreased to be moderate in group III (Fig. 5C) and mild in groups IV (Fig. 5D) and V (Fig. 5E). The mean value of NF-κB expression was the highest (5.29 ± 0.35) in group II whereas the lowest one was for group I (0.36 ± 0.18). The mean values for the NF-κB expression for groups III, IV, and V were 3.68 ± 0.24, 2.11 ± 0.19, and 0.62 ± 0.15, respectively. ANOVA test revealed a total significant difference between all groups (*P* = 0.001). Tukey’s post-hoc test revealed significant differences between group I and groups II, III and IV, between group II and groups III, IV, and V, between group III and groups IV and V (*P* = 0.001) and non-significant difference between group I and group V (*P* = 0.648) (Table 4; Fig. 5F).

## Discussion

Adjuvant-induced arthritis of animal models has been established for decades to study the pathogenesis of arthritis, including RA, gout, and OA, and to evaluate the effectiveness of certain anti-arthritic drugs^37^. Various CFA doses were tested to establish the arthritic models in earlier literature via IA, intradermal, or subcutaneous routes. In the present study, IA route was used as a reliable model for inducing arthritis and in line with ethical recommendations on reducing the severity of animal models of human disease. 50 µg CFA in 50 µl; on day 1 of the experiment was used to induce persistent inflammation and at day 14 to induce arthritis. This comes with Wang et al.^27^ who reported a novel model with double CFA injections into the upper compartment of the TMJ to induce structural abnormalities and degenerative changes in the disc and synovium. This contradicts other previous works that utilized only single CFA injections^38,39^ In addition, IA injection was the method used to deliver the therapeutic agents as it offers a localized and direct access to the joint space, thus facilitating the bioavailability of therapeutic agents at the affected site while reducing potential side effects, systemic exposure, and overall cost^40^. The authors speculated that local injection of the BMSCs-CM or its combination with HA will activate the endogenous repair mechanism by targeting the resident stem cells in the joint^41^. Therefore a single IA injection of BMSCs-CM or the combination could be considered as innovative formulation, well tolerated, safe, and effective in the treatment of TMJ OA.

The combination therapy in the present study had a better regenerative ability compared to HA hydrogel- or BMSCs-CM treated groups and we could attribute our results to the sustained release of the growth factors and cytokines found in the BMSCs-CM from the cross-linked HA hydrogel. Yang et al.^42^ achieved long-term retention of MSC-derived small extracellular vesicles in the joint area and controlled release by an injectable Diels-Alder cross-linked HA/PEG hydrogel for OA improvement. Similar to the findings of this study, Arifka et al.^43^ found that the embedding of MSC-CM in the HA hydrogel causes the discharge of cytokines/chemokines as well as anti- and/or pro-inflammatory molecules, and also overcomes the rapid removal of MSC-CM from the target tissue. The bioactivity of the CM contained in the gel is supported by in-vitro research allowing for a controlled and extended-release mechanism. In addition, Köhnke et al.^44^ reported that the addition of HA to mesenchymal stromal cells improve cartilage repair in a rabbit model of TMJ OA. In a beagle-dog model, Li et al.^45^ showed that BMSCs-CM in conjunction with HA had a more effective therapeutic impact than HA alone. Chiang et al.^46^ found that IA injections of allogeneic MSCs suspended in HA significantly slowed the course of osteoarthritis in rabbits compared to HA injections given singly. Huang et al.^47^ stated that MSCs-CM possess the capacity for both immune control and tissue healing. The therapeutic potential of MSCs-CM is improved by hydrogel encapsulation by boosting survival, retention, and targeting. The therapeutic effectiveness of hydrogel-loaded MSCs-CM has been established in a variety of illnesses, primarily in the regeneration of bone and cartilage. In addition, Shoma Suresh et al.^48^ reported that MSC- CM’s characteristics can be retained even after being encapsulated into nanoparticles (NP) and merged into a hydrogel to create a composite. Biocompatibility was shown by the MSC-CM hydrogel composite. Moreover, the NP-hydrogel composite’s regulated release of MSC-CM increased metabolic activity, highlighting the potential for regenerative medicine. Fabricating hydrogel encapsulation of MSCs-CM will have several advantages likely as sustained drug release effect, higher biocompatibility, ability to simulate extracellular-like conditions and can be scaled up in bulk not needing MSC expansion each time^49^.

HA hydrogel and BMSCs-CM have been well-identified as promising therapeutic agents to treat symptoms of arthritis. Local delivery of cross-linked HA hydrogel incorporated with BMSCs-CM was the method of administration in the present study. Localized route is considered more clinically relevant as it has several advantages over the systemic one. Local delivery increases bioavailability, reduce toxicity and side effects, and minimize off-target activity. As repetitive injections of arthritic TMJ with the medications could induce damage to the joint, cross-linked HA hydrogel was used to achieve a more sustained release of BMSCs-CM over a longer period of time. However developing a new medication in the form of HA hydrogel combined with BMSCs-CM still faces several challenges. On the one hand, is to determine the pharmacokinetic, pharmacodynamic properties and target identification of HA hydrogel when combined with BMSCs-CM. On the other hand, validation and optimization of this new medication requires more researches in the form of preclinical studies before human testing. Preclinical validation in the form of in-vitro and in-vivo trials are required to determine a starting, safe dose for first-in-human study and assess potential toxicity of the medication. After that randomized, placebo-controlled clinical trials should be designed to learn if this new medication is more effective or has less harmful side effects than existing treatments.

## Conclusion

It could be concluded that to repair degenerative changes in rats’ TMJs associated with CFA-induced arthritis, combination therapy of HA hydrogel and BMSCs-CM is better than using HA hydrogel or BMSCs-CM, separately.

## Data Availability

The data produced and analyzed for this study are available from the corresponding author upon reasonable request.

## References

[1] Lippi, L. et al. . Multidisciplinary rehabilitation after hyaluronic acid injections for elderly with knee, hip, shoulder, and temporomandibular joint osteoarthritis. *Medicina (Kaunas). ***59** (11), 2047 (2023). 10.3390/medicina59112047 PMC10672933 38004096

[2] Jameson, J. L. Harrison’s principles of internal medicine. McGraw-Hill education; 20th ed. New York, NY: (2018).

[3] Sodhi, A., Naik, S., Pai, A. & Anuradha, A. Rheumatoid arthritis affecting temporomandibular joint. *Contemp. Clin. Dent. ***6** (1), 124–127 (2015). 25684928 10.4103/0976-237X.149308 PMC4319332

[4] Izawa, T., Hutami, I. R. & Tanaka, E. Potential role of Rebamipide in Osteoclast differentiation and mandibular condylar cartilage homeostasis. *Curr. Rheumatol. Rev. ***14** (1), 62–69 (2018). 29046162 10.2174/1573397113666171017113441 PMC5925868

[5] Mohammed, A. et al. A comparison of risk factors for osteo- and rheumatoid arthritis using NHANES data. *Prev. Med. Rep. ***20**, 101242 (2020). 33294313 10.1016/j.pmedr.2020.101242 PMC7689317

[6] Liu, X. H. et al. Recent advances in enzyme-related biomaterials for arthritis treatment. *Front. Chem. ***10**, 988051 (2022). 36051622 10.3389/fchem.2022.988051 PMC9424673

[7] Lespasio, M., Mont, M. & Guarino, A. Identifying risk factors associated with postoperative infection following elective lower-extremity total joint arthroplasty. *Perm J. ***24**, 1–3 (2020). 33482967 10.7812/TPP/20.013 PMC7931988

[8] Migliore, A., Giovannangeli, F., Granata, M. & Laganà, B. Hylan g-f 20: review of its safety and efficacy in the management of joint pain in osteoarthritis. *Clin. Med. Insights Arthritis Musculoskelet. Disord. ***3**, 55–68 (2010). 21151854 PMC2998981

[9] Huynh, A. & Priefer, R. Hyaluronic acid applications in ophthalmology, rheumatology, and dermatology. *Carbohydr. Res. ***489**, 107950 (2020). 32070808 10.1016/j.carres.2020.107950

[10] Testa, G. et al. Intra-articular injections in knee osteoarthritis: a review of literature. *J. Funct. Morphol. Kinesiol. ***6** (1), 15 (2021). 33546408 10.3390/jfmk6010015 PMC7931012

[11] Fallacara, A., Baldini, E., Manfredini, S. & Vertuani, S. Hyaluronic acid in the third millennium. *Polymers (Basel). ***10** (7), 701 (2018). 10.3390/polym10070701 PMC6403654 30960626

[12] Newberry, S. J. et al. *Systematic Review for Effectiveness of Hyaluronic acid in the Treatment of Severe Degenerative Joint Disease (DJD) of the knee * (Agency for Healthcare Research and Quality (US), 2015). 26866204

[13] Kay, A. G. et al. Mesenchymal stem cell-conditioned medium reduces disease severity and Immune responses in inflammatory arthritis. *Sci. Rep. ***7** (1), 18019 (2017). 29269885 10.1038/s41598-017-18144-w PMC5740178

[14] Rosochowicz, M. A., Lach, M. S., Richter, M., Suchorska, W. M. & Trzeciak, T. Conditioned medium - is it an undervalued lab Waste with the potential for osteoarthritis management? *Stem Cell. Rev. Rep. ***19** (5), 1185–1213 (2023). 36790694 10.1007/s12015-023-10517-1 PMC10366316

[15] Veronesi, F. et al. The use of cell conditioned medium for musculoskeletal tissue regeneration. *J. Cell. Physiol. ***233** (6), 4423–4442 (2018). 29159853 10.1002/jcp.26291

[16] Li, L. et al. Conditioned medium from human adipose-derived mesenchymal stem cell culture prevents UVB-induced skin aging in human keratinocytes and dermal fibroblasts. *Int. J. Mol. Sci. ***21** (1), 49 (2019). 31861704 10.3390/ijms21010049 PMC6981944

[17] Smolinská, V., Boháč, M. & Danišovič, Ľ. Current status of the applications of conditioned media derived from mesenchymal stem cells for regenerative medicine. *Physiol. Res. ***72** (S3), S233–S245 (2023). 37888967 10.33549/physiolres.935186 PMC10669946

[18] Pang, K., Kong, F. & Wu, D. Prospect of mesenchymal stem-cell-conditioned medium in the treatment of acute pancreatitis: a systematic review. *Biomedicines. ***11** (9), 2343 (2023). 37760784 10.3390/biomedicines11092343 PMC10525511

[19] Jia, X. Y., Jing, S. L., Sun, Y., Gong, Z. C. & Guo, Z. C. A randomized controlled clinical trial of concentrated growth factor combined with sodium hyaluronate in the treatment of temporomandibular joint osteoarthritis. *BMC Oral Health. ***24** (1), 540 (2024). 38720327 10.1186/s12903-024-04258-x PMC11080079

[20] Hegab, A. F., Hameed, H. I. A. A., Hassaneen, A. M. & Hyder, A. Synergistic effect of platelet rich plasma with hyaluronic acid injection following arthrocentesis to reduce pain and improve function in TMJ osteoarthritis. *J. Stomatol. Oral Maxillofac. Surg. ***124** (1S), 101340 (2023). 36414172 10.1016/j.jormas.2022.11.016

[21] Xia, L. et al. Conditioned medium from stem cells of human exfoliated deciduous teeth alleviates mouse osteoarthritis by inducing sFRP1-expressing M2 macrophages. *Stem Cells Transl. Med. ***13** (4), 399–413 (2024). 38366885 10.1093/stcltm/szae006 PMC11016837

[22] Simental-Mendía, M. et al. Anti–inflammatory and anti–catabolic effect of non–animal stabilized hyaluronic acid and mesenchymal stem cell–conditioned medium in an osteoarthritis coculture model. *Mol. Med. Rep. ***21** (5), 2243–2250 (2020). 32323772 10.3892/mmr.2020.11004

[23] Noh, A. S. M. et al. Effects of different doses of complete Freund’s adjuvant on nociceptive behaviour and inflammatory parameters in polyarthritic rat model mimicking rheumatoid arthritis. *PLoS ONE. ***16** (12), e0260423 (2021). 34879087 10.1371/journal.pone.0260423 PMC8654228

[24] Gurram, S. et al. Amelioration of experimentally induced inflammatory arthritis by intra-articular injection of visnagin. *Curr. Res. Pharmacol. Drug Discov. ***3**, 100114 (2022). 35992378 10.1016/j.crphar.2022.100114 PMC9389203

[25] Kadam, P. & Bhalerao, S. Sample size calculation. *Int. J. Ayurveda Res. ***1** (1), 55–57 (2010). 20532100 10.4103/0974-7788.59946 PMC2876926

[26] Serdar, C. C., Cihan, M., Yücel, D. & Serdar, M. A. Sample size, power and effect size revisited: simplified and practical approaches in pre-clinical, clinical and laboratory studies. *Biochem. Med. (Zagreb). ***31** (1), 010502 (2021). 33380887 10.11613/BM.2021.010502 PMC7745163

[27] Wang, X. D., Kou, X. X., Mao, J. J., Gan, Y. H. & Zhou, Y. H. Sustained inflammation induces degeneration of the temporomandibular joint. *J. Dent. Res. ***91** (5), 499–505 (2012). 22427270 10.1177/0022034512441946 PMC3327731

[28] Zaki, A. A., Zaghloul, M., Helal, M. E., Mansour, N. A. & Grawish, M. E. Impact of autologous bone marrow-derived stem cells on degenerative changes of articulating surfaces associated with the arthritic temporomandibular joint: an experimental study in rabbits. *J. Oral Maxillofac. Surg. ***75** (12), 2529–2539 (2017). 28576669 10.1016/j.joms.2017.05.001

[29] Lemos, G. A., Rissi, R., Pimentel, E. R. & Palomari, E. T. Effects of high molecular weight hyaluronic acid on induced arthritis of the temporomandibular joint in rats. *Acta Histochem. ***117** (6), 566–575 (2015). 26022645 10.1016/j.acthis.2015.05.003

[30] Bousnaki, M. et al. Managing temporomandibular joint osteoarthritis by dental stem cell secretome. *Stem Cell. Rev. Rep.* (2023). 10.1007/s12015-023-10628-9 PMC10661765 37751010

[31] Liu, F. et al. Hyaluronic acid hydrogel integrated with mesenchymal stem cell-secretome to treat endometrial injury in a rat model of Asherman’s syndrome. *Adv. Healthc. Mater. ***8** (14), e1900411 (2019). 31148407 10.1002/adhm.201900411 PMC7045702

[32] Xu, L. et al. A time-dependent degeneration manner of condyle in rat CFA-induced inflamed TMJ. *Am. J. Transl. Res. ***8** (2), 556–567 (2016). 27158347 PMC4846904

[33] Pritzker, K. P. et al. Osteoarthritis cartilage histopathology: grading and staging. *Osteoarthr. Cartil. ***14** (1), 13–29 (2006). 10.1016/j.joca.2005.07.014 16242352

[34] Gnecchi, M. & Melo, L. G. Bone marrow-derived mesenchymal stem cells: isolation, expansion, characterization, viral transduction, and production of conditioned medium. *Methods Mol. Biol. ***482**, 281–294 (2009). 19089363 10.1007/978-1-59745-060-7_18

[35] Xin, Y. et al. Human foreskin-derived dermal stem/progenitor cell-conditioned medium combined with hyaluronic acid promotes extracellular matrix regeneration in diabetic wounds. *Stem Cell. Res. Ther. ***12** (1), 49 (2021). 33422138 10.1186/s13287-020-02116-5 PMC7796620

[36] Jiao, K. et al. Age- and sex-related changes of mandibular condylar cartilage and subchondral bone: a histomorphometric and micro-CT study in rats. *Arch. Oral Biol. ***55** (2), 155–163 (2010). 20034609 10.1016/j.archoralbio.2009.11.012

[37] Pandey, S. Various techniques for the evaluation of anti arthritic activity in animal models. *J. Adv. Pharm. Technol. Res. ***1** (2), 164–171 (2010). 22247842 PMC3255441

[38] Lai, Y. C. et al. Intraarticular induction of interleukin-1beta expression in the adult mouse, with resultant temporomandibular joint pathologic changes, dysfunction, and pain. *Arthritis Rheum. ***54** (4), 1184–1197 (2006). 16572453 10.1002/art.21771

[39] Flake, N. M., Hermanstyne, T. O. & Gold, M. S. Testosterone and estrogen have opposing actions on inflammation-induced plasma extravasation in the rat temporomandibular joint. *Am. J. Physiol. Regul. Integr. Comp. Physiol. ***291** (2), R343–R348 (2006). 16469833 10.1152/ajpregu.00835.2005

[40] Rai, M. F. & Pham, C. T. Intra-articular drug delivery systems for joint diseases. *Curr. Opin. Pharmacol. ***40**, 67–73 (2018). 29625332 10.1016/j.coph.2018.03.013 PMC6015522

[41] Zhang, X. Y. & He, D. M. Research progress in mesenchymal stem cell-related therapies in the treatment of temporomandibular joint osteoarthritis. *Zhonghua Kou Qiang Yi Xue Za Zhi. ***59** (7), 732–737 (2024). 38949143 10.3760/cma.j.cn112144-20230817-00097

[42] Yang, Y. et al. Controlled release of MSC-derived small extracellular vesicles by an injectable diels-Alder crosslinked hyaluronic acid/PEG hydrogel for osteoarthritis improvement. *Acta Biomater. ***128**, 163–174 (2021). 33862283 10.1016/j.actbio.2021.04.003

[43] Arifka, M., Wilar, G., Elamin, K. M. & Wathoni, N. Polymeric hydrogels as mesenchymal stem cell secretome delivery system in biomedical applications. *Polymers (Basel). ***14** (6), 1218 (2022). 10.3390/polym14061218 PMC8955913 35335547

[44] Köhnke, R. et al. Temporomandibular Joint Osteoarthritis: regenerative treatment by a stem cell containing advanced therapy medicinal product (ATMP)-An in vivo animal trial. *Int. J. Mol. Sci. ***22** (1), 443 (2021). 33466246 10.3390/ijms22010443 PMC7795212

[45] Li, L. et al. Mesenchymal stem cells in combination with hyaluronic acid for articular cartilage defects. *Sci. Rep. ***8** (1), 9900 (2018). 29967404 10.1038/s41598-018-27737-y PMC6028658

[46] Chiang, E. R. et al. Allogeneic mesenchymal stem cells in combination with hyaluronic acid for the treatment of osteoarthritis in rabbits. *PLoS ONE. ***11** (2), e0149835 (2016). 26915044 10.1371/journal.pone.0149835 PMC4767225

[47] Huang, Y., Li, X. & Yang, L. Hydrogel encapsulation: taking the therapy of mesenchymal stem cells and their derived secretome to the next level. *Front. Bioeng. Biotechnol. ***10**, 859927 (2022). 35433656 10.3389/fbioe.2022.859927 PMC9011103

[48] Shoma Suresh, K., Bhat, S., Guru, B. R., Muttigi, M. S. & Seetharam, R. N. A nanocomposite hydrogel delivery system for mesenchymal stromal cell secretome. *Stem Cell. Res. Ther. ***11** (1), 205 (2020). 32460846 10.1186/s13287-020-01712-9 PMC7251860

[49] Khayambashi, P. et al. Hydrogel encapsulation of mesenchymal stem cells and their derived exosomes for tissue engineering. *Int. J. Mol. Sci. ***22** (2), 684 (2021). 33445616 10.3390/ijms22020684 PMC7827932

